# The electric double layer effect and its strong suppression at Li^+^ solid electrolyte/hydrogenated diamond interfaces

**DOI:** 10.1038/s42004-021-00554-7

**Published:** 2021-08-06

**Authors:** Takashi Tsuchiya, Makoto Takayanagi, Kazutaka Mitsuishi, Masataka Imura, Shigenori Ueda, Yasuo Koide, Tohru Higuchi, Kazuya Terabe

**Affiliations:** 1 International Center for Materials Nanoarchitectonics (WPI-MANA), National Institute for Materials Science (NIMS), Tsukuba, Ibaraki Japan; 2 Department of Applied Physics, Faculty of Science, Tokyo University of Science, Katsushika, Tokyo Japan; 3 Research Center for Advanced Measurement and Characterization, NIMS, Tsukuba, Ibaraki Japan; 4 Research Center for Functional Materials, NIMS, Tsukuba, Ibaraki Japan; 5 Synchrotron X-ray Station at SPring-8, NIMS, Sayo, Hyogo Japan; 6 Research Network and Facility Services Division, NIMS, Tsukuba, Ibaraki Japan

**Keywords:** Batteries, Electronic devices, Batteries

## Abstract

The electric double layer (EDL) effect at solid electrolyte/electrode interfaces has been a key topic in many energy and nanoelectronics applications (e.g., all-solid-state Li^+^ batteries and memristors). However, its characterization remains difficult in comparison with liquid electrolytes. Herein, we use a novel method to show that the EDL effect, and its suppression at solid electrolyte/electronic material interfaces, can be characterized on the basis of the electric conduction characteristics of hydrogenated diamond(H-diamond)-based EDL transistors (EDLTs). Whereas H-diamond-based EDLT with a Li-Si-Zr-O Li^+^ solid electrolyte showed EDL-induced hole density modulation over a range of up to three orders of magnitude, EDLT with a Li-La-Ti-O (LLTO) Li^+^ solid electrolyte showed negligible enhancement, which indicates strong suppression of the EDL effect. Such suppression is attributed to charge neutralization in the LLTO, which is due to variation in the valence state of the Ti ions present. The method described is useful for quantitatively evaluating the EDL effect in various solid electrolytes.

## Abstract (web summary)

The effect of the electric double layer with solid electrolytes remains hard to characterize. In this study, the authors show how to evaluate the electric double layer effect with various lithium solid electrolytes using a hydrogenated diamond-based transistor.

## Introduction

Solid electrolyte-based electrochemical devices, or solid-state ionic devices, have attracted attention for use in a wide range of applications, from energy devices (e.g., all-solid-state lithium ion batteries(ASS-LIBs), capacitors, sensors) to information and communication technology (ICT) devices (e.g., memristors, atomic switch, neuromorphic devices, physical property tuning devices)^1–13^. While various bulk properties of solid electrolytes, including ionic conductivity and phase stability, have been intensively investigated to identify ways of improving the performance of such devices with a view to their practical use, recent reports indicated that not only the bulk properties but also the electric double layer (EDL) (or space charge layer) have an effect, which is due to ionic carrier density modulation in the vicinity of electrode/solid electrolyte playing a crucially important role in the device operation^10–13^. An instance, related to ASS-LIBs, of EDL formation accompanied by Li^+^ depletion has been discussed in relation to high interfacial resistance for Li^+^ transport near electrode/Li^+^ conducting sulfide electrolyte (e.g., Li_3.25_Ge_0.25_P_0.75_S_4_, 70Li_2_S—30P_2_S_5_) interfaces^10,11^. In an alternative discussion, the EDL effect was proposed to have very strong impact on the various switching properties and neuromorphic functionalities of electrochemical metallization devices (memristors, atomic switches) that are composed of metallic ion conducting oxide thin films (e.g., SiO_2_, Ta_2_O_5_)^12^.

While, until the present, the EDL effect in such solid state ionic devices has been one of the key issues, one additional important question needs to be asked; Does the EDL effect, which is accompanied by significant variation in ion concentration and the resultant huge capacitance of several µF/cm^2^ in liquid electrolyte systems^14–17^, occur even in solid electrolyte systems with rigid frameworks? While the importance of the clarification (e.g., extent of ion concentration variation, existence of induced charge on electrode surface, thickness of the EDL) is widely understood, direct observation of the EDL in the vicinity of solid/solid electrolytes interface has been difficult even by state-of-art transmission electron microscope (TEM) technologies. It is because, analogous to the EDL for liquid electrolytes, the EDL for solid electrolytes is expected to be extremely thin (e.g., in nm order) and extent of ion concentration variation is small. Furthermore, compared with liquid electrolytes, the excess charge of ions (anion/cation) in the EDL of solid electrolytes, in particular of inorganic oxides, is expected to be more readily neutralized by the additional electronic carrier doped in the solid electrolytes and the resultant redox state modulation of the component ions (e.g., Ti^4+^ to Ti^3+^)^18,19^. Such electrochemical charge neutralization (compensation) inside solid electrolytes can potentially deteriorate EDL capacitance greatly. However, the evaluation of the EDL capacitance in solid electrolytes is not straightforward because, in conventional electrochemical analyses, based on passing electric currents through electrolytes, such an electrochemical charge neutralization (i.e., chemical capacitance) can, in general, be observed with a large capacitance^20,21^, which in turn makes the evaluation of EDL capacitance difficult. Therefore, a novel approach is required to clarify EDL behavior in solid electrolyte systems.

Here, we report on our investigations into EDL-induced electronic charge accumulation, and its suppression at solid/solid interfaces by using EDL transistors (EDLTs) composed of hydrogenated (H-) diamond homoepitaxial film with (100)-oriented surface and Li^+^ conducting solid electrolyte thin films. Chemically stable H-diamond (100) homoepitaxial film was chosen to function as an ion-blocking channel (i.e., Li^+^ is not transferred between H-diamond and electrolytes) in EDLTs in order to detect electrostatic carrier doping due to the EDL effect by ruling out the redox effect on channel conductivity modulation^13–15^. The combination of the idealistic ion-blocking property of diamond and the Hall measurement, which is sensitive only to electronic carriers located on the surface of diamond (i.e., insensitive to electronic carriers in electrolytes) evidenced that the EDL effect, with huge EDL capacitance comparable to that found in liquid electrolytes, is achieved even with inorganic solid electrolytes. The results further indicated that inclusion of redox active elements, even if such elements are included only near the interface, suppresses EDL capacitance by several orders of magnitude. In situ hard x-ray photoelectron spectroscopy detected that a steep potential drop (several MV/cm) in sub-nm to nm-thick region with Li^+^ depletion formed at the ion-blocking electrode/LSZO interface under dc voltage applied conditions. Furthermore, in situ scanning transmission electron microscope (STEM)–electron energy loss spectroscopy (EELS) observation was performed to investigate EDL suppression mechanism. The present technique is useful for revealing EDL behavior in the vicinity of solid/solid electrolyte interfaces and for clarifying the effect of the interface characteristics on the total performance of solid state ionic devices, including ASS-LIBs.

## Results and discussion

### Hall measurement of H-diamond-based EDLTs consisting of Li^+^ electrolyte thin films

**Fig. 1:**
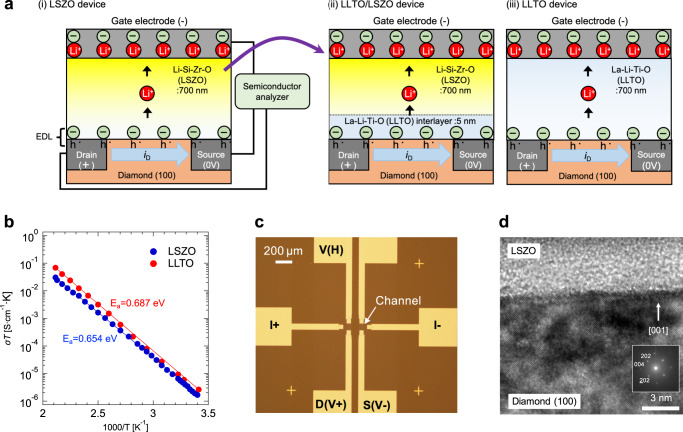
H-diamond-based EDLTs consisting of Li^+^ electrolyte thin films. **a** Illustrations of three diamond-based EDLTs consisted of Li-Si-Zr-O (LSZO) or La-Li-Ti-O (LLTO) Li^+^ solid electrolyte thin films [(i)LSZO device, (ii)LLTO/LZSO device, (iii)LLTO device]. Li^+^ in the electrolytes is depicted only for indicating polarity of electric current. **b** Arrhenius type plots of the ionic conductivities of LSZO and LLTO films. **c** Optical image of a hall-bar shaped hydrogen-terminated channel. **d** High resolution transmission electron microscope (HR-TEM) image of the LSZO/H-diamond interface.

Figure 1a illustrates diamond-based EDLTs consisting of Li-Si-Zr-O (LSZO) or La-Li-Ti-O (LLTO) Li^+^ solid electrolyte thin films, which were used to investigate the possibility of the charge neutralization effect occurring in the electrolyte on EDL formation^22–26^. In the subject three devices [(i)LSZO device, (ii)LLTO/LZSO device, (iii)LLTO device], the only difference is the electrolyte film used. LSZO and LLTO exhibited comparable conductivities (5.7 and 8.9 × 10^−9^ S/cm at RT) and activation energies (0.654 and 0.687 eV), as clarified by the Arrhenius type plots of ionic conductivities shown in Fig. 1b, whereas the compositions of the amorphous electrolytes are different, that is, only LLTO includes redox active Ti ions. Figure 1c shows an optical image of a hall-bar shaped hydrogen-terminated channel (500-μm-long and 800-μm-wide, with the channel indicated with an arrow), which was fabricated on the H-diamond surface by electron-beam lithography. The high resolution transmission electron microscope (HR-TEM) image of the LSZO/H-diamond interface shown in Fig. 1d confirms well crystalized H-diamond (100) and completely amorphized LSZO. For the LLTO/LSZO device, a 5-nm-thick interlayer was inserted at the LSZO/H-diamond interface. Li^+^/hole mixed conducting LiCoO_2_ (LCO) and Pt thin films were deposited as a gate electrode.

**Fig. 2:**
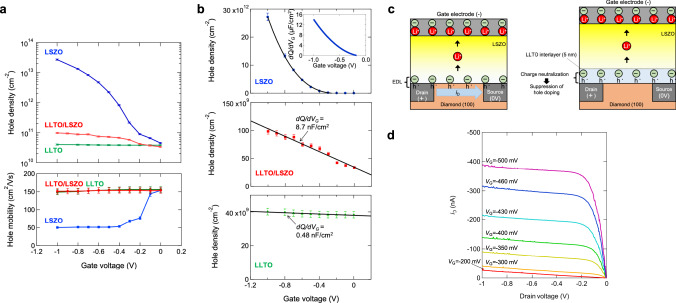
Hall measurement of H-diamond-based EDLTs. **a** Variation in hole density (upper panel) and hole mobility (lower panel) for H-diamond (100)-based EDLTs consisting of LSZO, LLTO/LSZO, and LLTO electrolytes determined by Hall measurements. **b** Linear plot of the variation in hole density shown in Fig. 2a. **c** Illustrations of the difference in hole density modulation behaviors in LSZO and LLTO/LSZO devices. Li^+^ in the electrolytes is depicted only for indicating polarity of electric current. **d** *i*_D_ vs. *V*_D_ characteristic of the LSZO device. Error bars in the figure represent SD.

The upper panel in Fig. 2a shows variation in hole density for H-diamond (100)-based EDLTs consisting of LSZO, LLTO/LSZO, and LLTO electrolytes measured by Hall measurement. The EDLT consisting of LSZO, hereinafter referred to as the LSZO device, showed a hole density increase of three order of magnitude as the gate voltage (*V*_G_) was tuned in a narrow voltage range of from 0 to −1 V. Note that *V*_G_ application in negative polarity triggers Li^+^ transport from the H-diamond side to the LCO gate electrode side. By taking account of the excellent chemical stability of diamond under ambient conditions (e.g., RT, atmospheric pressure)^27^, increase in hole density due to Li^+^ insertion/desertion and the resultant variation in electronic carrier density (i.e., redox) can be excluded. Furthermore, electronic conductivity in oxide electrolyte films (e.g., LSZO, LLTO) is negligibly low with respect to that in H-diamond with very high hole mobility (>50 cm^2^/V). The increase in hole density in Fig. 2a is thus attributed to hole doping in two-dimensional hole gas (2DHG) of the H-diamond surface via EDL formation in the vicinity of the H-diamond/LSZO Li^+^ electrolyte interface. For the LSZO device, a steep decrease in hole mobility from 150 to 50 cm^2^/Vs was observed, as shown in the lower panel of Fig. 2a. This is due to enhanced phonon scattering, which is a main scattering mechanism for electronic conduction in H-diamond 2DHG near RT. Similar behavior has been reported for H-diamond-based EDLTs composed of liquid electrolytes^17^.

For the LSZO device, the maximum hole density, 2.7 × 10^13^ cm^−2^, is slightly higher (by 35%) than that reported for H-diamond (100)-based EDLTs with ionic liquid, 2 × 10^13^ cm^−2^ ^17^. Furthermore, as indicated in the inset of Fig. 2b, EDL capacitance (*C*_EDL_) at the H-diamond/LSZO interface is calculated to be up to 14 μF/cm^2^, which is far larger than the 2.1 μF/cm^2^ found in liquid type EDLTs^17^. This is determined by the variation in hole density in the *V*_G_ region from −0.5 to −1.0 V, by considering the relationship $\frac{{dQ}}{{dV_G}} = C_{EDL}\left( {{{{{{{{\mathrm{differential}}}}}}}}\,{{{{{{{\mathrm{capacitance}}}}}}}}\,{{{{{{{\mathrm{of}}}}}}}}\,{{{{{{{\mathrm{EDL}}}}}}}}} \right)$, which has been widely used for the characterization of EDL^28–30^. Since the relationship is valid only when no depression layer is formed in the channel related to a contribution from depression layer capacitance (thus the validity is dependent on *V*_G_ with semiconductor channels as in the present case), the very low differential capacitance observed below −0.3 V is attributed not to a pure *C*_EDL_ but to a combined capacitance (a serial combination) of *C*_EDL_ and depression layer capacitance of the diamond channel, which can be far lower than *C*_EDL_ (please refer to Supplementary discussion 1. for the details). Although the origin of the difference between this study and EDLT with a liquid electrolyte^17^ is not clear at present, the large *C*_EDL_ at the large negative *V*_G_ is advantageous for steep slope FET applications^31^. The origin of *C*_EDL_ in the LSZO device was confirmed to be Li^+^ in LSZO by comparison with a reference device consisted of Si-Zr-O dielectric film (without Li^+^) in additional experiment. Please refer to Supplementary discussion 2 for the details^32^.

Additional EDLTs were fabricated to enable investigation of the possibility of the presence of a charge neutralization effect inside solid electrolyte; One such EDLT consisted of LSZO with a 5-nm-thick interlayer of La-Li-Ti-O (LLTO) Li^+^ solid electrolyte, the other consisted only of LLTO, as illustrated in Fig. 1a. These devices are hereinafter denoted the LLTO/LSZO device and the LLTO device, respectively. While the LSZO device exhibited a three orders of magnitude enhancement in hole density with large EDL capacitance up to 14 μF/cm^2^, as shown in Fig. 2a, b, the LLTO/LSZO device showed a very small enhancement, from 3.4 to 9.8 × 10^10^ cm^−2^ (in Fig. 2a), with extremely small differential capacitance, 8.7 nF/cm^2^ (in Fig. 2b), which is three orders of magnitude lower than the LSZO device and thus an unrealistic value for EDL capacitance. Furthermore, carrier doping behavior was completely absent in the LLTO device, as seen in Fig. 2a, b. The significant reduction of the carrier doping effect and small capacitance for the LLTO/LSZO and LLTO devices evidences that hole doping due to EDL formation is suppressed in the vicinity of the LLTO/H-diamond interface. The origin of the suppression is attributed to variation in the oxidation state of Ti ions, between trivalent and quadravalent states, which is frequently seen in various oxide materials^18,33,34^. Figure 2c illustrates the different behavior of the devices discussed above. Note that the LLTO/electrode interface have a good contact, which was clarified by STEM observation (please refer to Supplementary discussion 3 for a quality of the LLTO/electrode contact). Therefore, we could rule out a possibility that a poor contact at the LLTO/electrode interface caused the significant difference between LSZO and LLTO devices. In the following section, said behavior will be discussed in the framework of defect chemistry. Note that the significant suppression of EDL charging with 5-nm-thick LLTO layer indicates EDL in LSZO device occurs in the range of 5 nm from the LSZO/diamond interface. Given counter charges of the maximum hole density (2.7 × 10^13^ cm^−2^) are distributed in the range of the LSZO, the density of negatively-charged Li^+^ vacancy is calculated to be 1.8% of Li^+^ in LSZO.

To further investigate the similarity of LSZO devices to normal dielectric-based FETs, the drain current (*i*_D_) vs. drain voltage (*V*_D_) characteristic of the LSZO device was investigated, as shown in Fig. 2d. The clear pinch off behavior, consisting of both a linear region in small *V*_D_ and saturation in large *V*_D_, similar to that usually seen with normal dielectric-based FETs, indicates that the EDLT conducts electrostatic carrier doping. These similarities to EDLTs using normal FETs and liquid electrolytes strongly supports our contention that our EDLT operates with an EDL mechanism regardless of the type of electrolyte, that is, liquid or solid.

Studies have shown that proton conduction occurs in H-containing solid electrolytes in which protons are mobile, and thus work as electric carriers^35,36^. On the other hand, the hydrogen found on the surface of H-diamond is known to be tightly bonded with carbon atoms in the diamond^37^, making the mobility of the protons extremely low. Therefore, given that the present study uses H-diamond, protons does not work as electric carriers and have no effect on the formation of the electric double layer.

### Interpretation of the device characteristics based on defect chemistry

**Fig. 3:**
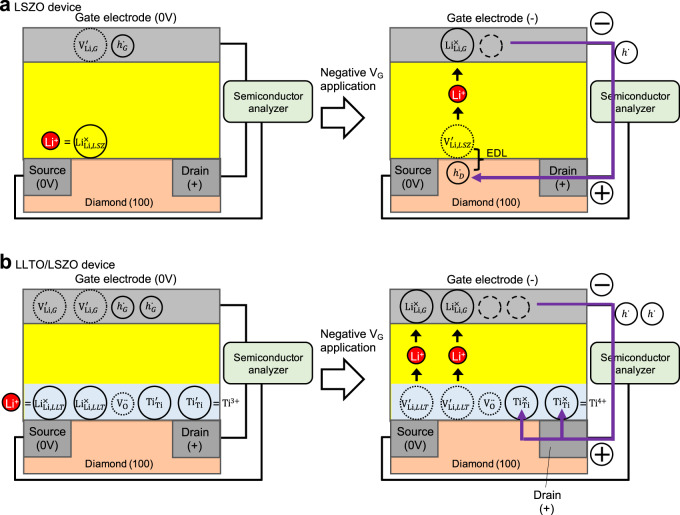
Interpretation of the device characteristics based on defect chemistry. Illustrations of EDL formation in LSZO device **a** and the suppression in LLTO/LSZO device **b** under the negative *V*_G_ applied condition [the polarity at all terminals is the same as shown in Figs. 1a and 2c]. Li^+^ in the electrolyte and $h^ \cdot$ in the lead wire are shown only as indicators of the polarity of the electric current.

In the following, carrier doping mechanisms are discussed in detail, with the help of defect chemistry, to clarify the differences in characteristics between LSZO and LLTO/LSZO devices. In case of the LSZO device, because of the ion-blocking nature of H-diamond (i.e., Li^+^ is not supplied from H-diamond), negative charge is formed due to Li^+^ removal from the interface, which is driven by *V*_G_ application. Left and right panels in Fig. 3(a) schematically show the behavior of charge carriers, which can be expressed using Kröger–Vink notation, as follows:

1 $${{{{{{{\mathrm{Li}}}}}}}}_{{{{{{{{\mathrm{Li}}}}}}}},\,LSZ}^ \times + {{{{{{{\mathrm{V}}}}}}}}_{{{{{{{{\mathrm{Li}}}}}}}},\,G}^\prime + h_G^ \cdot \to {{{{{{{\mathrm{V}}}}}}}}_{{{{{{{{\mathrm{Li}}}}}}}},\,LSZ}^\prime + h_D^ \cdot + {{{{{{{\mathrm{Li}}}}}}}}_{{{{{{{{\mathrm{Li}}}}}}}},\,G}^ \times$$

${{{{{{{\mathrm{Li}}}}}}}}_{{{{{{{{\mathrm{Li}}}}}}}},\,LSZ}^ \times$, ${{{{{{{\mathrm{V}}}}}}}}_{{{{{{{{\mathrm{Li}}}}}}}},\,G}^\prime$, $h_G^ \cdot$, ${{{{{{{\mathrm{V}}}}}}}}_{{{{{{{{\mathrm{Li}}}}}}}},\,LSZ}^\prime$, $h_D^ \cdot$, and ${{{{{{{\mathrm{Li}}}}}}}}_{{{{{{{{\mathrm{Li}}}}}}}},\,G}^ \times$ are Li^+^ in LSZO near the H-diamond/LSZO interface, negatively-charged Li^+^ vacancy in the LCO gate (*G*), hole in the LCO gate (*G*), negatively-charged Li^+^ vacancy in LSZO near the H-diamond/LSZO interface, hole doped in H-diamond (*D*), and Li^+^ transferred from the H-diamond interface to the LCO gate (*G*), respectively. In the initial state [the left-hand side of Eq. (1), depicted in the left panel in Fig. 3a], ${{{{{{{\mathrm{V}}}}}}}}_{{{{{{{{\mathrm{Li}}}}}}}},\,G}^\prime \,and\,h_G^ \cdot$ are neutralized in the LCO gate. By Li^+^ transport from the H-diamond/LSZO interface to the LCO gate, as shown in the right-hand side of Eq. (1) [depicted in the right panel in Fig. 3a], a hole with a positive charge is doped in the H-diamond ($h_D^ \cdot$) through EDL formation with negatively-charged ${{{{{{{\mathrm{V}}}}}}}}_{{{{{{{{\mathrm{Li}}}}}}}},\,LSZ}^\prime$ in the LSZO in the vicinity of the H-diamond/LSZO interface. The above discussion confirms that electrostatic carrier doping due to EDL formation requires ${{{{{{{\mathrm{V}}}}}}}}_{{{{{{{{\mathrm{Li}}}}}}}},\,LSZ}^\prime$ in LSZO and that, if the excess charge is neutralized inside LSZO $( {{{{{{{{\mathrm{V}}}}}}}}_{{{{{{{{\mathrm{Li}}}}}}}},\,LSZ}^\prime + h_{LSZ}^ \cdot = {{{{{{{\mathrm{V}}}}}}}}_{{{{{{{{\mathrm{Li}}}}}}}},\,LSZ}^ \times } )$, modulation of $h_D^ \cdot$ is not obtained.

Note that the part of the expression using Kröger–Vink notation for the amorphous electrolytes [e.g., Eq. (1)] is an approximation because definition of point defects is strictly valid only for crystalline structures^12^. Even in cases with SiO_2_ based amorphous materials, various types of defects, including oxygen deficiency centers, dangling bonds, and interstitial oxygen, are known to work as point defects with positive/negative site charges^12^. In the present case, ${{{{{{{\mathrm{V}}}}}}}}_{{{{{{{{\mathrm{Li}}}}}}}},\,LSZ}^\prime$ in LSZO is assumed to be a dangling bond (e.g., Si-O^2−^) formed by Li^+^ removal.

On the other hand, the situation changes for the LLTO/LSZO device. Left and right panels in Fig. 3b schematically show the behavior of charge carriers, in the same manner as in Fig. 3a, which can be expressed as follows:

2 $$2{{{{{{{\mathrm{Li}}}}}}}}_{{{{{{{{\mathrm{Li}}}}}}}},\,LLT}^ \times + 2{{{{{{{\mathrm{Ti}}}}}}}}_{{{{{{{{\mathrm{Ti}}}}}}}}}^\prime + {{{{{{{\mathrm{V}}}}}}}}_{{{{{{{\mathrm{O}}}}}}}}^{ \cdot \cdot } + {{{{{{{\mathrm{V}}}}}}}}_{{{{{{{{\mathrm{Li}}}}}}}},\,G}^\prime + h_G^ \cdot \to 2{{{{{{{\mathrm{V}}}}}}}}_{{{{{{{{\mathrm{Li}}}}}}}},\,LLT}^\prime + 2{{{{{{{\mathrm{Ti}}}}}}}}_{{{{{{{{\mathrm{Ti}}}}}}}}}^ \times + {{{{{{{\mathrm{V}}}}}}}}_{{{{{{{\mathrm{O}}}}}}}}^{ \cdot \cdot } + 2{{{{{{{\mathrm{Li}}}}}}}}_{{{{{{{{\mathrm{Li}}}}}}}},\,G}^ \times$$

${{{{{{{\mathrm{Li}}}}}}}}_{{{{{{{{\mathrm{Li}}}}}}}},\,LLT}^ \times ,\,{{{{{{{\mathrm{Ti}}}}}}}}_{{{{{{{{\mathrm{Ti}}}}}}}}}^\prime$, ${{{{{{{\mathrm{V}}}}}}}}_{{{{{{{\mathrm{O}}}}}}}}^{ \cdot \cdot }$, and ${{{{{{{\mathrm{Ti}}}}}}}}_{{{{{{{{\mathrm{Ti}}}}}}}}}^ \times$ are Li^+^ in the LLTO interlayer, Ti^3+^ in the LLTO interlayer (inherent with oxygen deficiency), oxygen vacancy, and Ti^4+^ in the LLTO layer respectively. In the present case, point defect approximation, similar to that for LSZO discussed with Eq. (1), is applied to LLTO (e.g., ${{{{{{{\mathrm{Li}}}}}}}}_{{{{{{{{\mathrm{Li}}}}}}}},\,LLTO}^ \times ,\,{{{{{{{\mathrm{Ti}}}}}}}}_{{{{{{{{\mathrm{Ti}}}}}}}}}^\prime$, ${{{{{{{\mathrm{V}}}}}}}}_{{{{{{{\mathrm{O}}}}}}}}^{ \cdot \cdot }$, ${{{{{{{\mathrm{Ti}}}}}}}}_{{{{{{{{\mathrm{Ti}}}}}}}}}^ \times$).

In the initial state [the left-hand side of Eq. (2), depicted in the left panel in Fig. 3b], excess charges of ${{{{{{{\mathrm{Ti}}}}}}}}_{{{{{{{{\mathrm{Ti}}}}}}}}}^\prime$ and ${{{{{{{\mathrm{V}}}}}}}}_{{{{{{{\mathrm{O}}}}}}}}^{ \cdot \cdot }$, which are inherent as oxygen non-stoichiometry, neutralize each other in LLTO. By applying negative *V*_G_, Li^+^ transport creates negatively charged Li^+^ vacancies (e.g., ${{{{{{{\mathrm{V}}}}}}}}_{{{{{{{{\mathrm{Li}}}}}}}},\,LLT}^\prime$ in LLTO) near the H-diamond/LLTO interface, as shown in the right-hand side of Eq. (2) [depicted in the right panel in Fig. 3b]. However, contrary to the case for the LSZO device in which such Li^+^ vacancy forms EDL with a hole in H-diamond, valence variation in Ti ions from Ti^3+^ (${{{{{{{\mathrm{Ti}}}}}}}}_{{{{{{{{\mathrm{Ti}}}}}}}}}^\prime$) to Ti^4+^ (${{{{{{{\mathrm{Ti}}}}}}}}_{{{{{{{{\mathrm{Ti}}}}}}}}}^ \times$) makes the negative charge of the Li^+^ vacancy (${{{{{{{\mathrm{V}}}}}}}}_{{{{{{{{\mathrm{Li}}}}}}}},\,LLTO}^\prime$) compensate with a positive charge of oxygen vacancy (${{{{{{{\mathrm{V}}}}}}}}_{{{{{{{\mathrm{O}}}}}}}}^{ \cdot \cdot }$) in the LLTO interlayer, thus preventing electrostatic hole doping in H-diamond.

When comparing the two cases, it is evident that, with the LSZO device, holes are doped in H-diamond under negative *V*_G_ application conditions, while it is doped in the LLTO interlayer with the LLTO/LSZO device. Since the hole doping corresponds to a lowering of the chemical potential of electrons, μ_e-_, the difference between them can be determined by the location of the μ_e-_ modulation. Whereas μ_e-_ is modulated in H-diamond for the LSZO device, it is modulated in the LLTO interlayer for the LLTO/LSZO device. To generalize the difference, the EDL effect appears in solid electrolytes in which only μ_Li+_ is modulated, while it is absent in solid electrolytes in which both μ_Li+_ and μ_e-_ are modulated.

### Comparison with a LiCoO_2_-based redox transistor

**Fig. 4:**
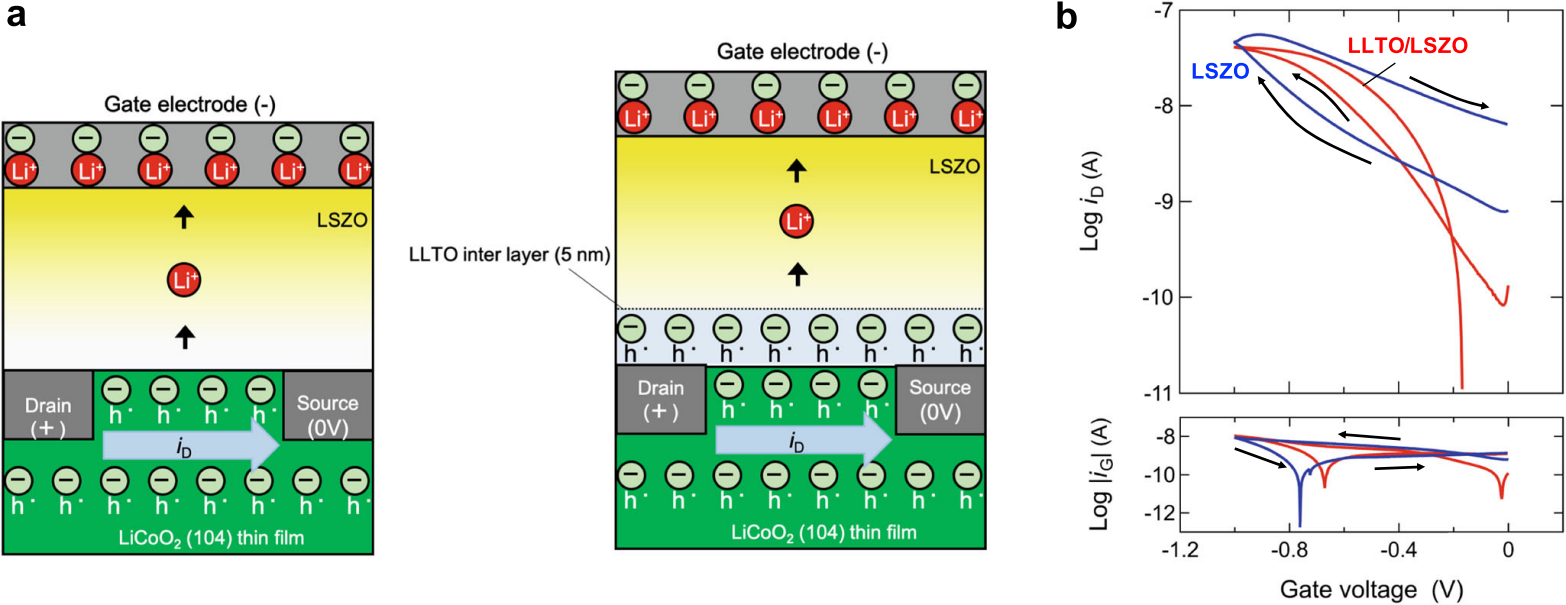
Comparison with a LiCoO_2_-based redox transistor. **a** Illustrations of the two LCO-based transistors consisting of LSZO or LSZO/LLTO electrolytes. Li^+^ in the electrolytes shown only as indicators of the polarity of the electric current. **b** *i*_D_ vs. *V*_G_ (upper panel) and *i*_G_ vs. *V*_G_ (lower panel) characteristic of the devices.

As discussed above, the distinct difference in hole density variation between the LSZO and LSZO/LLTO devices strongly indicates that Ti ion redox in the LLTO interlayer plays a crucially important role in the neutralization of EDL charges, however, another possibility, the interruption of Li^+^ transport by the LLTO interlayer, cannot be completely ruled out. In order to clarify the role of the LLTO interlayer, namely (i) EDL charge neutralization or (ii) interruption of Li^+^ transport, we fabricated LSZO and LSZO/LLTO devices in which only the H-diamond component was replaced by LiCoO_2_ (LCO) thin film. Figure 4a illustrates the two fabricated LCO-based transistors, incorporating LSZO or LSZO/LLTO electrolytes. LCO is known to be a hole and Li^+^ mixed conductor, in which Li^+^ (and electron) removal is accompanied by hole doping^34^.

3 $${{{{{{{\mathrm{Li}}}}}}}}_{{{{{{{{\mathrm{Li}}}}}}}}}^ \times + {{{{{{{\mathrm{Co}}}}}}}}_{{{{{{{{\mathrm{Co}}}}}}}}}^ \times \to {{{{{{{\mathrm{Li}}}}}}}}^ + + e\prime + {{{{{{{\mathrm{V}}}}}}}}_{{{{{{{{\mathrm{Li}}}}}}}}}^\prime + {{{{{{{\mathrm{Co}}}}}}}}_{{{{{{{{\mathrm{Co}}}}}}}}}^ \cdot$$

A Li^+^ vacancy on the Li site and a positive charge on the Co site work as acceptor and hole, respectively. Therefore, the predominant electric (hole) conductivity can be controlled *in-situ* by varying the Li^+^ vacancy concentration (lithium non-stoichiometry) in the LCO; LCO-based transistors operate as redox transistors, as seen in recent memristive applications^38,39^.

Compared to the case with H-diamond, Hall measurement of LCO-based transistors is rather difficult due to low electronic hole mobility in LCO and the resultant small Hall voltage. In order to observe hole density variation, drain current (*i*_D_) variation was measured instead, although that was a somewhat indirect approach. The upper-panel in Fig. 4b shows *i*_D_ variation for LCO-based redox transistors consisting of LSZO or LSZO/LLTO electrolyte thin films. *i*_D_ for the two transistors gradually increased as *V*_G_ was tuned from 0 to 1 V. The *i*_D_ increase is well understood as increase in hole density due to redox reactions in LCO, as shown in Eq. (3) (toward the right-hand side). The significant gate current shown in the lower panel of Fig. 4b also supports the redox mechanism. However, contrary to what was observed in the case of the H-diamond EDLTs shown in Fig. 2, the maximum hole density (corresponding to the maximum *i*_D_), observed at *V*_G_ = −1 V for the LCO-based LSZO and LSZO/LLTO devices, was almost identical. The result shows that similar amounts of hole and Li^+^ vacancies were introduced in LCO with or without the LLTO interlayer. In other words, (ii) the interruption of Li^+^ transport discussed above can be ruled out as having a role in the LLTO interlayer. As seen in the Arrhenius-type plots of the Li^+^ conductivity of LZSO and LLTO thin films shown in Fig. 1b, the very close Li^+^ conductivities of LSZO and LLTO (i.e., 5.7 and 8.9 × 10^−9^ S/cm at room temperature) are consistent with our assumption that the LLTO interlayer (5 nm thick) in the LSZO thin film (700 nm thick) has no significant effect on Li^+^ transport from the H-diamond/Li^+^ electrolyte interface to the gate electrode in the devices.

### In situ STEM-EELS observation at a diamond electrode/LLTO interlayer interface under a dc voltage applied condition

**Fig. 5:**
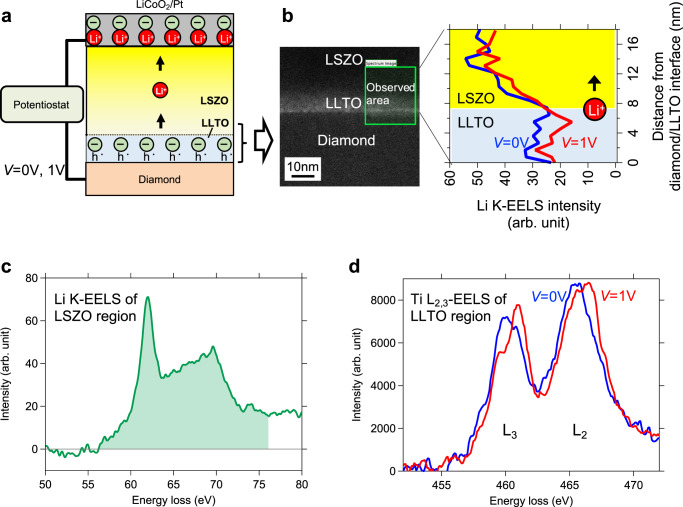
In situ STEM-EELS observation at a diamond electrode/LLTO interlayer interface. **a** Illustration of the two terminal cell with a diamond working electrode, an LSZO electrolyte, and an LCO/Pt counter/reference electrode for in situ STEM-EELS. Li^+^ in the electrolytes is shown only as indicators of the polarity of the electric current. **b** TEM image near the diamond/LLTO interlayer interface (left panel) and the depth profile of the Li K-edge intensity under dc voltage (*V*=0 V, 1 V) applied conditions (right panel). **c** Li K-edge spectrum measured in the LSZO region. Background subtraction and zero loss deconvolution were applied to a raw spectrum in order to remove any plural scattering effect and to obtain the single scattering distribution shown in Fig. 5c. **d** Ti L-edge spectra measured under 0 V and 1 V applied conditions.

In order to clarify mechanism for the strong suppression of the EDL effect based on (i) EDL charge neutralization due to the redox of Ti ions (as depicted in Fig. 3b), we performed in situ STEM-EELS (Li-K and Ti-L edges) observations in the vicinity of the diamond/LLTO interlayer interface with the two terminal cell illustrated in Fig. 5a. To secure sufficient electrical conductance in the diamond electrode, boron doped diamond was used instead of non-doped diamond. The left-hand panel of Fig. 5b shows a TEM image of the area near the diamond/LLTO interlayer interface under 0 V applied condition. The observed area is indicated by a green square. Figure 5c shows the Li K-EEL spectrum measured in the LSZO region under 0 V applied condition. The spectrum shape is similar to the ones for LiF and LiPF_6_, which are used as salts for the electrolytes in Li ion batteries^40,41^. By integration of the Li K-EEL spectrum [the colored region from 56 to 76 eV in the Fig. 5c] with respect to the distance from the diamond/LLTO interlayer interface, we calculated the depth profile of the Li K-EELS integrated intensity, which is equivalent to the Li ion concentration, under dc voltage applied conditions (*V* = 0 V, 1 V), as shown in the right-hand panel of Fig. 5b. A significant decrease in the Li K-EELS integrated intensity was observed in both the LLTO interlayer and the LSZO region upon application of 1 V to the diamond electrode, in which Li^+^ is removed from the vicinity of the interface due to Li^+^ transport towards the LCO/Pt electrode. Specifically, a decrease of about 20% was observed in the LLTO interlayer. These results are in good agreement with the discussion on Fig. 4, that is, the LLTO interlayer is not interrupting Li^+^ transport and the Li^+^ concentration thus changes in the LLTO interlayer.

Figure 5d shows the Ti L-EEL spectra measured under 0 V and 1 V applied conditions. Under both conditions, Ti L_2_ and L_3_ peaks, which are located at about 466 and 460 eV respectively, are observed, while the two peaks show a shift to the higher energy side upon 1 V application. Ti L_2_ and L_3_ peaks are known to show such chemical shift towards the higher energy side, by approximately 1 eV, as the oxidation state of Ti changes from Ti^3+^ to Ti^4+^ ^42^. As the peak shapes for L_2_ and L_3_ are considered to consist of energy loss peaks, mainly from Ti^4+^ with a possible contribution from Ti^3+^, the peak shift observed in both L_2_ and L_3_ is interpreted as a relative intensity Ti^4+^ peak increase under the 1 V application condition. Such behavior is interpreted as the redox of Ti ions from Ti^3+^ to Ti^4+^ in the LLTO interlayer caused by Li^+^ transport, and as such is consistent with the discussion in Fig. 3. Therefore, based on the discussion in Figs. 4 and 5, we conclude that the LLTO interlayer causes strong suppression of the EDL effect via (i) EDL charge neutralization, which is due to redox of Ti ions.

### Response speed of LSZO device at various temperatures

**Fig. 6:**
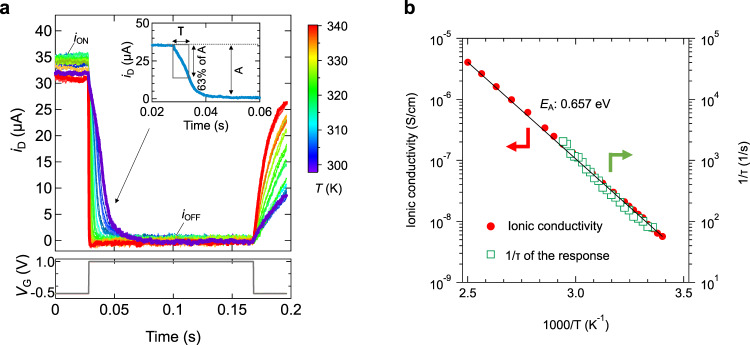
Response speed of LSZO device at various temperatures. **a** *i*_D_ vs. time characteristic of LSZO device, during single *V*_G_ pulse application, measured at various temperatures (298 to 340 K). **b** Comparison with 1/ *τ* (derived from Fig. 5a) and ionic conductivity of LSZO (derived from Fig. 1b) in Arrhenius type plots.

The switching response of EDLT is ideally a function of ionic conductivity in the electrolyte, based on the standard EDL charging mechanism, in which a serial connection of *C*_EDL_ and the resistance of the electrolyte (*R*_Electrolyte_) gives a time constant of the response (i.e., *τ* =*R*_Electrolyte_*C*_EDL_). In our study, to verify the validity of the EDL mechanism, we investigated the response behavior of the LSZO device under pulse *V*_G_ application conditions. Figure 6a shows *i*_D_ vs. time characteristic during single *V*_G_ pulse applications, measured at various temperature (298 to 340 K). Acceleration of the *i*_D_ switching response from the ON state (*V*_G_ = −0.5 V) to the OFF state (*V*_G_ = 1.0 V), observed in the time range from 0.03 to 0.06 s, is evident as the temperature is elevated, indicating that EDL charging becomes faster due to enhanced ionic conductivity of the LSZO at the elevated temperature. While the switching took several tens ms at 298 K, it took less than 1 ms at 340 K. To quantitatively discuss the relationship between the response and the ionic conductivity of the LSZO shown in Fig. 1b, the time constant (*τ*) is derived by considering the condition, where, at *t*= *τ*, *i*_D_ reduces to 1/e of the initial value A [*i.e*., 37% of A (decrement is 63% of A)] in the *RC* (*R*_Electrolyte_*C*_EDL_) circuit (regarded as a first order linear time-invariant system), as shown in Fig. 6a inset. Figure 6b shows a comparison of 1/ *τ* and ionic conductivity in Arrhenius type plots. As clearly seen in Fig. 6b, the two shows excellent agreement with each other over the whole temperature range, with an activation energy of 0.657 eV and an apparent *C*_EDL_ of 1.1 μF/cm^2^. The result evidences the standard EDL charging mechanism, approximated by Li^+^ transport in the LSZO and subsequent EDL charging at the LSZO/H-diamond interface, can be applied to the EDLT. The small value of apparent *C*_EDL_ is consistent with the variable capacitance shown in Fig. 2b, which becomes small below *V*_G_ of −0.3 V [overlapping to the main *V*_G_ range (−0.5 to 1.0 V) for the pulse measurement].

### In situ hard X-ray photoelectron spectroscopy observation at an Au electrode/LSZO interface under a dc voltage applied condition

**Fig. 7:**
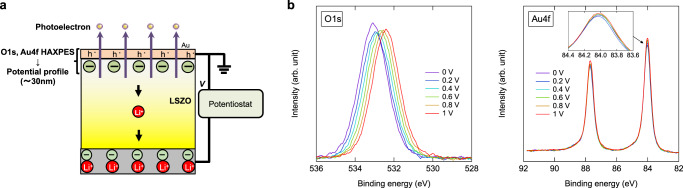
In situ hard X-ray photoelectron spectroscopy observation at the Au electrode/LSZO interface. **a** Illustration of the two terminal cell with Au top electrode, LSZO electrolyte, and LCO/Pt bottom electrode for in situ HAXPES. Li^+^ in the electrolytes is shown only as indicators of the polarity of the electric current. **b** O 1 s and **c** Au 4 f HAXPES spectra measured under various voltage applied conditions (0 to 1 V). Inset of **c** is a magnification of Au 4f_7/2_ peak.

The various electrochemical measurements shown above indicate ion blocking behaviors for LSZO solid electrolyte similar to the those seen in liquid electrolytes with EDL, accompanied by steep potential drops in sub-nm to nm-ordered-thick regions. In Li^+^ solid electrolytes, in which only Li^+^ is mobile, EDL can form not only in Li^+^ excess conditions but also in Li+ deficient conditions. This is due to the fact that once Li^+^ is reduced at the interface, a negative charge of anions (e.g. O^2−^) is left to form EDL with a high potential drop. Non-destructive, in situ hard X-ray photoelectron spectroscopy (HAXPES) was used to assess potential drop in the vicinity of the LSZO interface^8,9,43,44^. Figure 7a is an illustration of the two terminal cell for in situ HAXPES. Au film was used as the top electrode, since it can be fabricated safely onto an LSZO film surface and is known to be a typical Li^+^ blocking material^18,19,45^, instead of diamond, which is difficult to fabricate as a top electrode without causing serious degradation to the LSZO film. Except for the top electrode, all components of the cell, including LiCoO_2_/Pt electrode, were same as those of the LSZO-based EDLT shown in Figs. 1–6. Please refer to the figure captions for the experimental details. In situ O 1 s and Au 4 f HAXPES spectra are shown in Fig. 7b, c, measured with positive voltage applied to the Au top electrode. The O 1 s signal observed here is assigned to oxygen ions in the LSZO film beneath the Au top electrode. As positive voltage increased from 0 to 1 V, the O 1 s peak in Fig. 7b showed a significant peak shift toward the lower binding energy side and broadened slightly. Since the binding energy of the O1s photoelectron peak is varied by local electrostatic potential, if the potential profile in LSZO is not flat within the observed depth region, the shape of the observed O1s spectrum (the sum of O1s photoelectrons that emitted from the probing depth region) varies accordingly. Thus, the potential profile can be simulated based on the peak shape and the inelastic mean free path (IMFP) of the O 1s photoelectrons in LSZO. Please note that the contribution from in-plane potential drop to the O 1s peak shape variation can be ignored due to the absence in peak shape variation in Au 4 f spectra^9^, as shown as a function of applied voltage in Fig. 7c and inset.

**Fig. 8:**
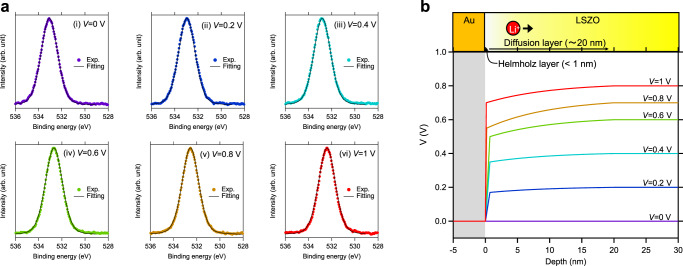
Simulated O 1 s spectra and potential profiles in the vicinity of the Au electrode/LSZO interface. **a** Simulated O 1 s HAXPES spectra. **b** Simulated potential profiles with respect to applied voltages.

We employed COMPRO software^46^ to simulate the potential profiles in the vicinity of the Au/LSZO film interface, by using the O 1 s spectra shapes and IMFP of O 1 s photoelectrons, which was calculated to be 8.4 nm for the kinetic energy of O 1 s (5420 eV), based on the TPP2M equation using the physical parameters of Zr-Si-O glass (density 4.6 g/cm^3^, molar mass 183.3 g/mol, and energy gap 6.5 eV)^47,48^. Please refer to Supplementary discussion 4 for information depth of the measurement. The O 1 s spectra simulated under various positive voltage application conditions (0 to 1 V) are shown with experimentally observed O 1 s spectra in Fig. 8a. The simulated O1s spectra agreed well with the experimentally observed O 1s spectra, supporting validity of the simulation. Figure 8b shows the potential profiles simulated under various positive voltage application conditions (0 to 1 V). Two regions are observed in the profiles; one has a very steep potential jump (e.g., several MV/cm) at the interface within a thickness of 0.13 to 0.7-nm from the interface, while the other has a relatively gentle slope (e.g., several tens kV/cm) within a thickness of 10 to 20-nm from the interface. The potential profiles are similar to the characteristic one found at the liquid electrolyte/electrode interface in the 2-layer-EDL model proposed by Stern and Grahame, which consisted of a densely charged Helmholz layer and a diffusion layer^49,50^. In their model, with its highly concentrated liquid electrolyte (e.g., 1 mmol/cm^3^), large voltage is applied to the extremely thin (0.2 to 0.4 nm) Helmholz layer,while the residual voltage is applied to the several-nm-thick diffusion layer^49,50^. It is quite reasonable to consider the two cases to be similar, given the high Li^+^ concentration (10 atomic% is equivalent to 2.4 mmol/cm^3^) in LSZO. The Helmholz layer thickness of 0.2 to 0.4 nm is equivalent to no more than one to two chemical bond lengths (e.g., Zr-O; 0.22 nm, Si-O; 0.16 nm), which indicates that a large voltage is applied between Au atoms, which are nearest to the LSZO, and the first nearest ions (e.g., oxygen, silicon, zirconium) in the LSZO. This is consistent with the steep potential jump at the Au/LSZO interface in Fig. 8b. As Li^+^ vacancy increases at the interface, caused by Li^+^ transport due to the applied voltage, it forms a sub-nm-thick Helmholz layer and a several-nm-thick diffusion layer, even when such inorganic solid electrolytes are used instead of liquid electrolytes.

## Conclusion

EDL-induced electronic carrier accumulation and its suppression at Li^+^ conducting solid electrolyte thin films/electronic material interfaces were investigated on the basis of the electric conduction characteristics of H-diamond-based EDLTs. Whereas significant modulation of hole density was observed for the LSZO device (accompanied by Li^+^ vacancy formation corresponding to 1.8% of Li^+^ in the LSZO), it was almost completely absent from the LLTO/LSZO and LLTO devices, indicating that redox of Ti ions suppresses EDL charging in the vicinity of the electrolyte/H-diamond interfaces. For the suppression mechanism, interruption of Li^+^ transport was ruled out by additional investigation of the electrical characteristic of LCO-based redox transistors, supporting our contention that neutralization of EDL charge prevents hole density modulation in the LLTO/LSZO device. The thermal activation behavior of the switching response in the LSZO device quantitatively agreed with that of the ionic conductivity of LSZO. The result confirms that the EDL charging mechanism with a steep potential drop near the electrode interface, observed by in situ HAXPES, applies to the all-solid-state EDLTs. Moreover, the EDL effect at solid electrolyte/electrode interfaces has been discussed relevant to the charging /discharging performance of all-solid batteries^10,11^. The present technique can be a powerful tool for revealing the EDL effect for various electrolytes by the quantitative evaluation of the charges, which has proved difficult to do by any other means.

## Methods

### Measurements

The electrical conduction characteristics of the transistors were evaluated using a 4200-scs semiconductor parameter analyzer, a 2182 A nanovoltmeter and a 6220 DC current source (Keithley, U.S.A.) in vacuum at RT. A dipole-type electromagnet (Tesla, Japan) was used to apply a magnetic field for Hall measurement. To cancel any undesired offset in measured Hall voltage, current pulses of +200 nA and −200 nA were applied alternately to the I^+^ and I^−^ electrodes (shown in Fig. 1c), and the Hall voltage was measured using V(H) and D(V+) electrodes (i.e. delta mode measurement) under various magnetic field application conditions. The Hall constant was calculated by using the slope of the Hall voltage vs. the applied magnetic field relationship obtained from the experiments. The intrinsic hole density of diamond was not subtracted from the calculated hole density in the measurement. For in situ STEM-EELS observation in the vicinity of the diamond/LLTO interlayer interface, JEM-ARM200F (JEOL, Japan) with EELS (Quantum, Gatan, USA) and FUSION special specimen holder for in situ measurements (Protochips, USA) was used.

The ionic conductivities for the electrolyte films (LSZO, LLTO) were measured by using AC impedance spectroscopy (1 MHz to 0.1 Hz, AC 30 mV) with the Pt/700-μm-thick electrolyte film/Pt two terminal cells. The AC impedance spectroscopy was also used for electrical property of the transistors. Please see Supplementary discussion 5 for AC impedance spectra of the devices.

### Fabrication of thin films and devices

First, 500-nm-thick H-diamond homoepitaxial film was deposited on the surface of an Ib-type high-pressure high-temperature (HPHT) diamond (100) single crystal (Element Six, Luxembourg)^22,23^. The deposition was done using microwave plasma chemical vapor deposition at 1213 K, with H_2_ and CH_4_ gas fluxes fixed at 1000 and 0.5 sccm, respectively. Please refer to the previous report for details of the fabrication^23^. Pd, Ti and Au thin films (10, 10, 200 nm thick, respectively) were deposited onto the surface, by electron beam evaporation, to form the six electrodes. Good ohmic contact was achieved by Pd insertion^24^. Subsequently, for the LSZO (LLTO) device, 700-nm-thick LSZO (LLTO) was deposited on the surface using pulsed laser deposition (PLD), using a 193-nm ArF excimer laser, which is advantageous for the efficient ablation of wide bandgap materials without droplet generation^25,26^. Li concentration in the LSZO and LLTO films were 9.0 and 4.8 atomic % measured by secondary ion mass spectrometry and nuclear reaction analysis.

## Supplementary information

Supplementary information

## Data Availability

The datasets generated during and/or analyzed during the current study are available from the corresponding author on reasonable request.
