# Supplementary material for: The electric double layer effect and its strong suppression at Li+ solid electrolyte/hydrogenated diamond interfaces
Source: Commun Chem. 2021 Aug 6;4:117. doi: 10.1038/s42004-021-00554-7 (PMC9814946; doi:10.1038/s42004-021-00554-7)
Supplement: Supplementary file 1 — Supplementary information [file 42004_2021_554_MOESM1_ESM.pdf]

Supplementary information

The Electric Double Layer Effect and its Strong Suppression at Li^+ Solid Electrolyte/Hydrogenated Diamond Interfaces

Takashi Tsuchiya[1*], Makoto Takayanagi[1,2], Kazutaka Mitsuishi[3], Masataka Imura[4], Shigenori Ueda[4,5], Yasuo Koide[6], Tohru Higuchi[2], and Kazuya Terabe[1]

[1]International Center for Materials Nanoarchitectonics (WPI-MANA), National Institute for Materials Science (NIMS), 1-1 Namiki, Tsukuba, Ibaraki, 305-0044, Japan.

[2]Department of Applied Physics, Faculty of Science, Tokyo University of Science, Katsushika, Tokyo 125-8585, Japan

[3]Research Center for Advanced Measurement and Characterization, NIMS, 1-2-1 Sengen, Tsukuba, Ibaraki, 305-0047, Japan.

[4]Research Center for Functional Materials, NIMS, 1-1 Namiki, Tsukuba, Ibaraki, 305-0044, Japan.

[5]Synchrotron X-ray Station at SPring-8, NIMS, 1-1-1 Kouto, Sayo, Hyogo 679-5148, Japan

[6]Research Network and Facility Services Division, NIMS, 1-2-1 Sengen, Tsukuba, Ibaraki, 305-0047, Japan.

[*]Email: TSUCHIYA.Takashi@nims.go.jp

Contents

Supplementary discussion

1. Energy diagrams and the equivalent circuits of the LSZO/diamond interface under forward and reverse bias conditions · 2
2. Comparisons of variation in hole density for H-diamond (100)-based transistors consisting of SZO, LSZO, LLTO/LSZO, and LLTO oxide films taken by Hall measurements · · · · · · · · · · · · 4
3. TEM and STEM observation of the LLTO/electrode interface · · · · · · · · · · · · · · · · · 6
4. Consideration of attenuation length of x-ray and photoelectron for in situ HAXPES · · · · · 7
5. AC impedance spectra measured with LSZO and LLTO/LSZO devices · · · · · · · · · · · · · 9

Supplementary discussion

1. Energy diagrams and the equivalent circuits of the LSZO/diamond interface under forward and reverse bias conditions

The large variation in the differential capacitance ($\frac{dQ}{dV_G}$) in Fig. 2**b** inset does not completely correspond to the one in C_{EDL}. It is because the relationship $\frac{dQ}{dV_G} = C_{EDL}(\text{differential capacitance of EDL})$ is valid only when no depression layer is formed in the channel related to a contribution from depression layer capacitance. So, the validity of the relationship $\frac{dQ}{dV_G} = C_{EDL}$ is dependent on V_G, with semiconductor channels as in the present case. Supplementary Figure 1a,b shows energy diagrams and the equivalent circuits of the LSZO/diamond interface under **a** forward and **b** reverse bias conditions. By considering a depression in semiconductor diamond channel under reverse bias condition(b), the very low differential capacitance observed below -0.3 V is attributed not to a pure C_{EDL} but to a combined capacitance (a serial combination) of C_{EDL} and depression layer capacitance (C_{DL}) of the diamond channel, which can be far lower than a pure C_{EDL}. Therefore, $\frac{dQ}{dV_G}$ only in the V_G range with high carrier density(<-0.5 V), corresponds to C_{EDL}. Please note that the reverse condition does not necessarily mean opposite V_G polarity in such an electrochemical system.

On the other hand, if the channel is consisted of a metallic material, such a depression in the channel does not occur. Supplementary Figure 1**c**,**d** shows energy diagrams and the equivalent circuits of the LSZO/metallic channel interface under **c** negative V_G and **d** positive V_G conditions, for comparison. In the case, the capacitance of the interface always equals to C_{EDL} regardless of V_G, as long as there is no other parasitic capacitance in the electrolyte. (But in this case, a detection of EDL charge by hall effect is difficult and hence the metallic channel is not useful for the purpose of the present study.)

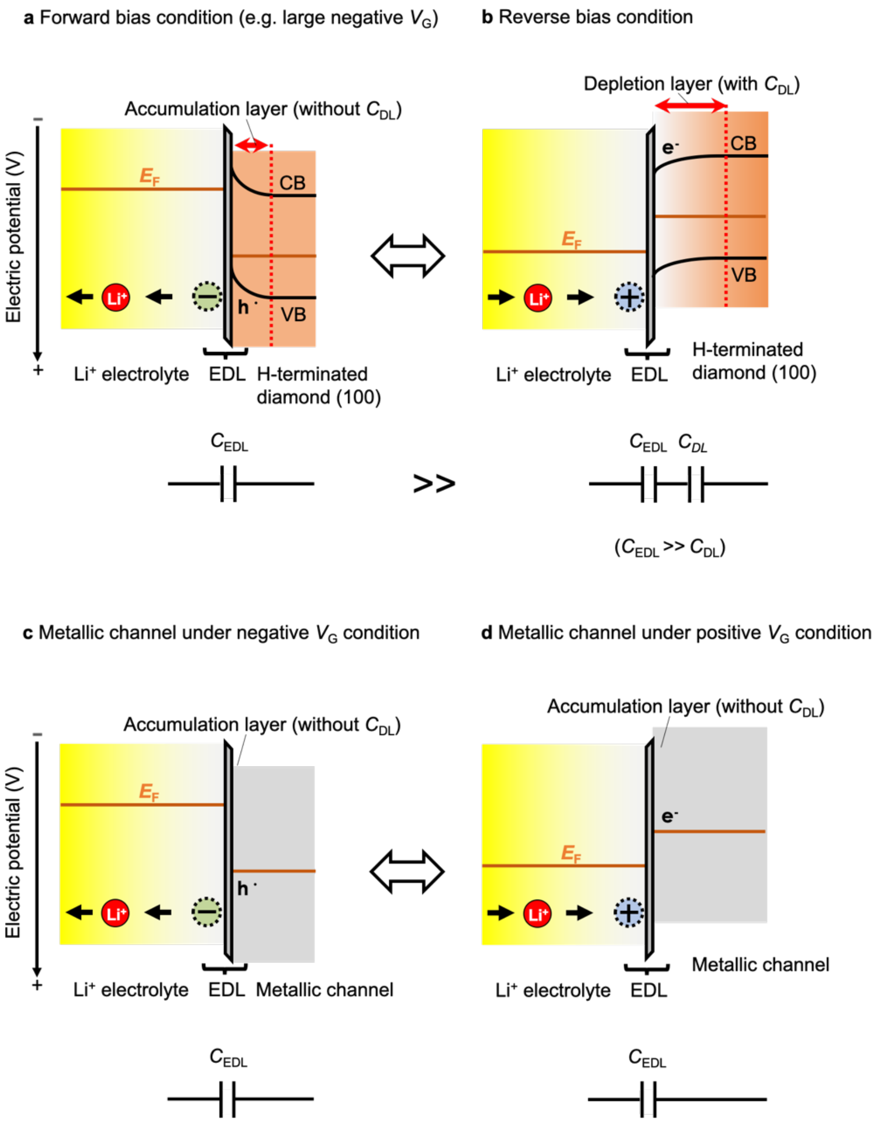

Supplementary Figure 1. Energy diagrams and the equivalent circuits of the LSZO/diamond interface under **a** forward and **b** reverse bias conditions. Energy diagrams and the equivalent circuits of the LSZO/metallic channel interface under **c** negative V_G and **d** positive V_G conditions.

2. Comparisons of variation in hole density for H-diamond (100)-based transistors consisting of SZO, LSZO, LLTO/LSZO, and LLTO oxide films taken by Hall measurements

We fabricated the diamond-based transistor illustrated in Supplementary Figure 2**a**. The transistor is identical to the LSZO device shown in Fig. 1**a**, except for the oxide film. In the present device, Si-Zr-O (SZO) amorphous thin film (700-µm-thick), which lacks Li^+, was used. Supplementary Figure 2**b** shows comparisons of variation in hole density for H-diamond (100)-based transistors consisting of SZO, LSZO, LLTO/LSZO, and LLTO oxide films taken by Hall measurements. As seen from the results, the SZO device exhibited very small hole density modulation within the V_G range, while the LSZO device showed significant hole density modulation. The difference is due to the absence of Li^+ in SZO, and the resultant low capacitance. From the result for SZO shown in Supplementary Figure 2**c**, the dielectric constant of SZO in the present study was calculated to be 6.8, which is close to the reported value (8.2) for a poly crystalline tetragonal SZO[1]. The deviation can be tentatively attributed to difference in the form of the SZO (amorphous SZO in our case), although the details of such attribution are not clear at present.

Based on the measurement results, SZO cannot achieve the significant hole density modulation observed in the LSZO device. Therefore, the present result clarifies the crucially important role that the mobile Li^+ in LSZO plays in modulating the hole density of the diamond channel.

a

b

c

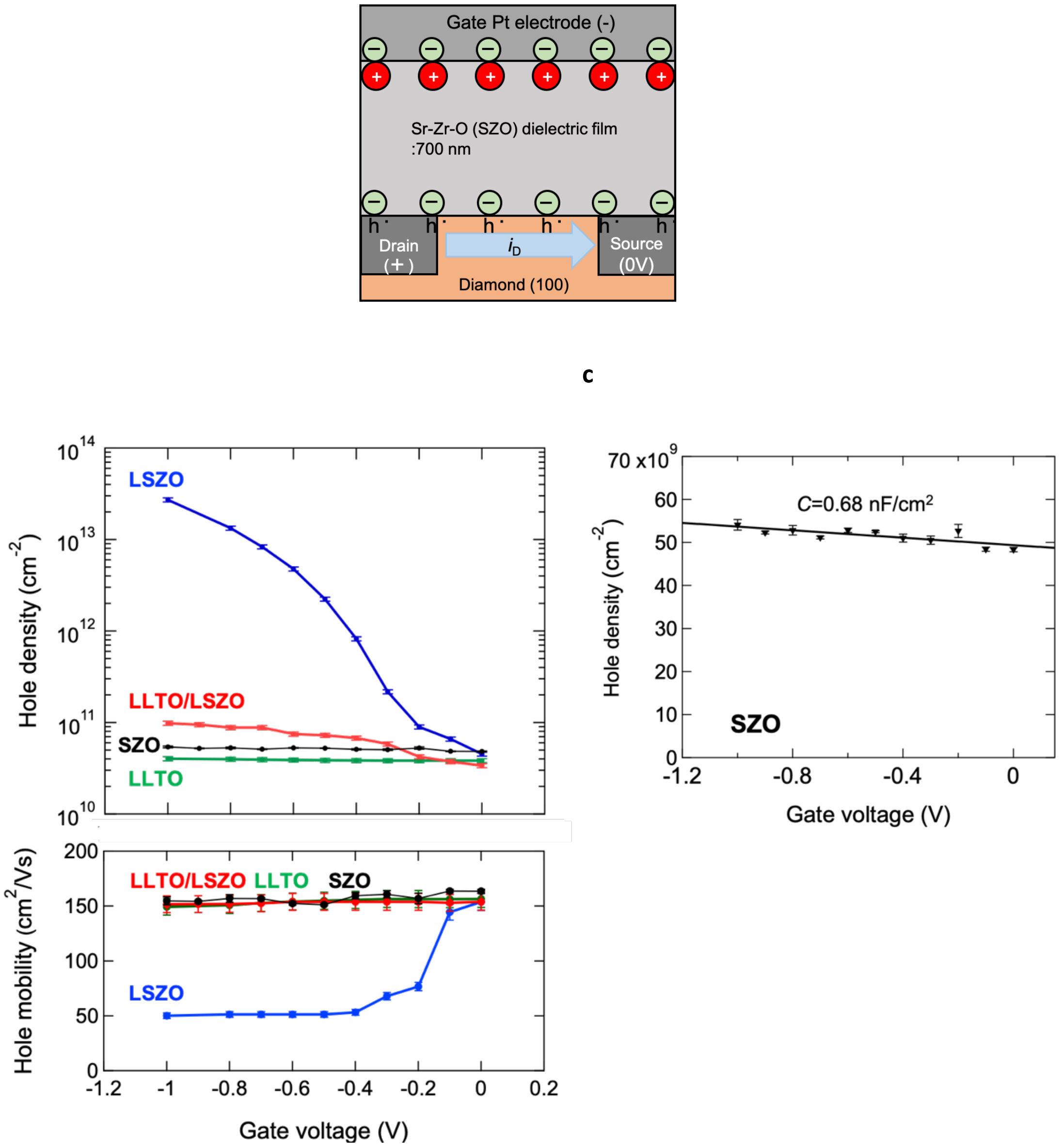

Supplementary Figure 2. **a** Illustration of diamond-based EDLTs consisting of Si-Zr-O (SZO) solid dielectric thin film. **b** Comparisons of variation in hole density for H-diamond (100)-based transistors consisting of SZO, LSZO, LLTO/LSZO, and LLTO oxide films taken by Hall measurements. **c** Linear plot of the variation in hole density for SZO device.

3. TEM and STEM observation of the LLTO/electrode interface

In order to rule out a possibility that a poor contact at the LLTO/electrode interface caused the significant difference between LSZO and LLTO devices, TEM and STEM observation was performed. Supplementary Figure 3**a,b** show low and high magnification cross section TEM images near the LLTO/electrode(consisted of Au with Ti/Pd insertion layer). In Supplementary Figure 3**a,b**, we could not find any part showing a peeling off between electrode interface. Furthermore, STEM observation was performed to search for micro or nano cracks between Au and LLTO. Supplementary Figure 3**c,d** shows low and high magnification STEM images near the LLTO/electrode interface. Even in the high magnification STEM image shown in Supplementary Figure 3**d**, we could not find any micro cracks near the interface, meaning that LLTO has a good contact with the electrode. Therefore, we concluded that the significant difference between LSZO and LLTO devices is note related to the quality of the electrode contact.

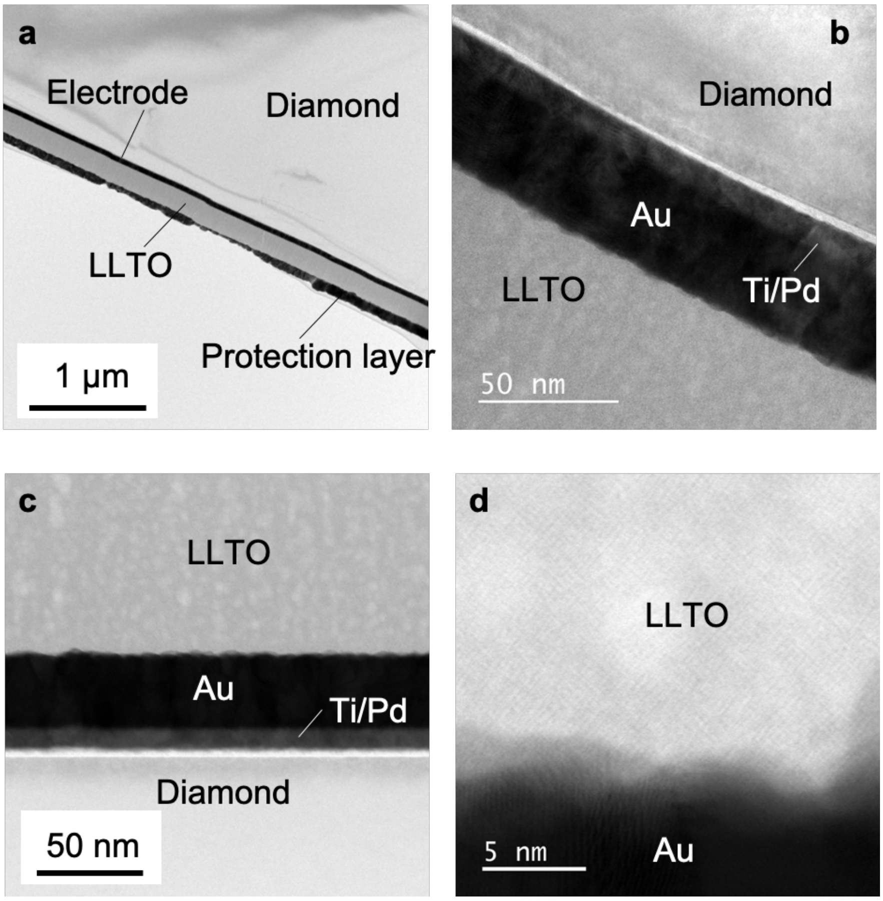

Supplementary Figure 3. **a** Low magnification and **b** high magnification cross section TEM images near the LLTO/electrode interface. **c** Low magnification and **d** high magnification cross section STEM images near the LLTO/electrode interface.

4. Consideration of attenuation length of x-ray and photoelectron for in situ HAXPES

In in situ HAXPES shown in Figs. 7 and 8, thickness of Au top electrode was 3 nm and attenuation depth of hard X-ray (5950 eV) in Au and Si-Zr-O[chosen instead of Li-Si-Zr-O (LSZO) because physical parameters needed for the calculation are available only for Si-Zr-O] are calculated to be 12 and 1.1 micron respectively, as indicated by black arrows in Supplementary Figure 4**a**. They are far longer than the thickness of Au top electrode (3 nm) and solid electrolyte oxide film (700 nm). So, in this study, the hard x-ray surely penetrates deep into the bottom electrode and the information depth of the HAXPES measurement is determined not by the attenuation depth of the used hard x-ray, but by the attenuation of photoelectrons.

Next, let us discuss on the attenuation of O1s photoelectrons emitted from LSZO by extending the above discussion. Supplementary Figure 4**b** shows inelastic mean free path (IMFP) of photoelectron in Si-Zr-O (for LSZO) and Au calculated, based on their physical parameters, as a function of kinetic energy of photoelectron. IMFP of O 1s photoelectron with a kinetic energy of 5420 eV[hard X-ray energy (5950 eV)- binding energy of O 1s (530 eV)] for Si-Zr-O and Au are obtained from Supplementary Figure 4**b** to be 8.4 and 4.6 nm, respectively. By applying these IMFP values to the Au/LSZO cell shown in Fig. 7**a**, relative intensity of O 1s photoelectron emitted from each depth is calculated as shown in Supplementary Figure 4**c**. Normalized intensity of O 1s photoelectron is attenuated during passing through LSZO. Although the intensity is maintained to 30% even at the depth of 10 nm owing to the used hard x-ray, it drops as low as 3% at the depth of 30 nm, supporting the O 1s photoelectron collected in the measurement is contributed mostly from the one above the depth of 30 nm. These attenuation characteristics of photoelectron under the present condition was used in the HAXPE spectra shape and potential profile simulation shown in Figs. 8**a,b**.

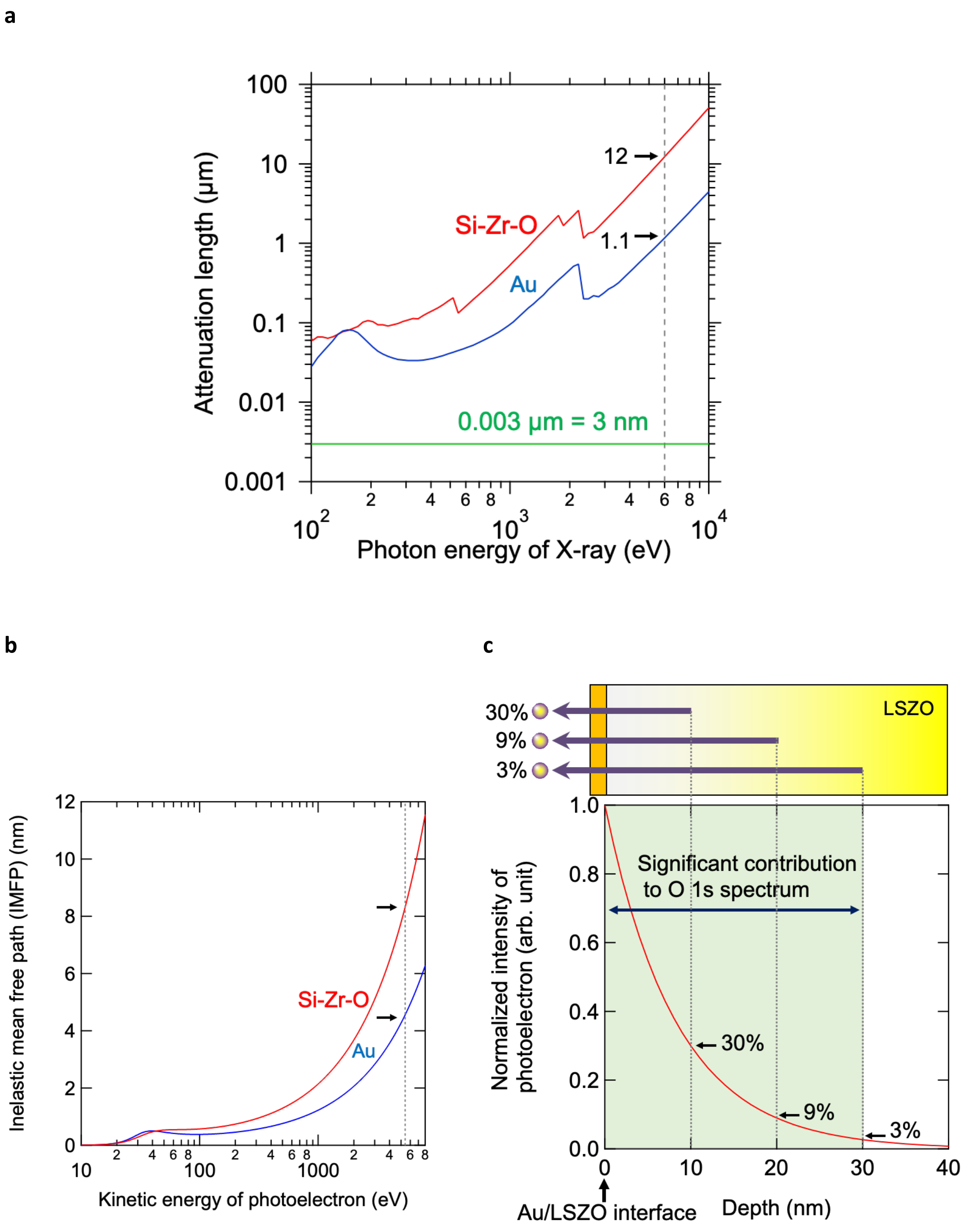

Supplementary Figure 4. **a** Calculated attenuation depth of X-ray in Au and Si-Zr-O. **b** IMFP of photoelectron in Si-Zr-O (for LSZO) and Au calculated as a function of kinetic energy of photoelectron. **c** Normalized intensity of O 1s photoelectron emitted from each depth.

5. AC impedance spectra measured with LSZO and LLTO/LSZO devices

Concerning the possibility of poor contact with LLTO, we have checked the alternating current (AC) impedance spectra shown in Supplementary Figure 5 measured with LSZO and LLTO/LSZO devices, which are shown in Fig. 1**a**. Said spectra were measured by applying AC 50 mV between the gate and drain electrodes. The distorted semicircle (indicated by a black arrow) is attributed to ionic transport impedance for the oxide electrolyte films(LSZO film for the LSZO device and LLTO/LSZO film for the LLTO/LSZO device, respectively). If the LLTO interface contact is poor, ionic transport impedance for the LLTO/LSZO device becomes large because the cross section of the ion transport path (LLTO/LSZO) is reduced. However, there was no difference in the ionic transport impedance between the LSZO and LLTO/LSZO devices. The result supports our understanding that the LLTO interface was not poor. In addition, we could find no evidence of poor LLTO contact during STEM observations of the diamond/LLTO interface shown in Fig. 5 and the electrode/LLTO interface shown in Supplementary Figure 3. Therefore, we conclude that any contribution from poor LLTO contact can be ruled out.

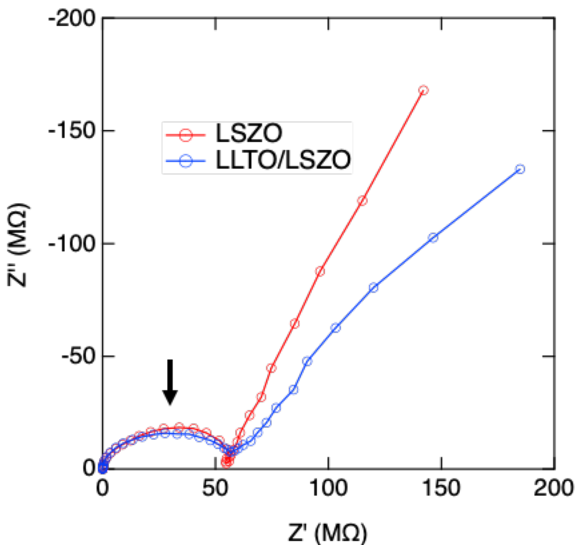

Supplementary Figure 5. AC impedance spectra measured with LSZO and LLTO/LSZO devices.

Reference

1. Neumayer, D.A. Cartier, E. Materials characterization of ZrO_2-SiO_2 and HfO_2-SiO_2 binary oxides deposited by chemical solution deposition. *J. Appl. Phys*. **90**, 1801(2001).
